# A novel stock forecasting model based on High-order-fuzzy-fluctuation Trends and Back Propagation Neural Network

**DOI:** 10.1371/journal.pone.0192366

**Published:** 2018-02-08

**Authors:** Hongjun Guan, Zongli Dai, Aiwu Zhao, Jie He

**Affiliations:** 1 School of Management Science and Engineering, Shandong University of Finance and Economics, Jinan, Shandong, China; 2 School of Management, Jiangsu University, Zhenjiang, Jiangsu, China; Jiangnan University, CHINA

## Abstract

In this paper, we propose a hybrid method to forecast the stock prices called High-order-fuzzy-fluctuation-Trends-based Back Propagation(HTBP)Neural Network model. First, we compare each value of the historical training data with the previous day's value to obtain a fluctuation trend time series (FTTS). On this basis, the FTTS blur into fuzzy time series (FFTS) based on the fluctuation of the increasing, equality, decreasing amplitude and direction. Since the relationship between FFTS and future wave trends is nonlinear, the HTBP neural network algorithm is used to find the mapping rules in the form of self-learning. Finally, the results of the algorithm output are used to predict future fluctuations. The proposed model provides some innovative features:(1)It combines fuzzy set theory and neural network algorithm to avoid overfitting problems existed in traditional models. (2)BP neural network algorithm can intelligently explore the internal rules of the actual existence of sequential data, without the need to analyze the influence factors of specific rules and the path of action. (3)The hybrid modal can reasonably remove noises from the internal rules by proper fuzzy treatment. This paper takes the TAIEX data set of Taiwan stock exchange as an example, and compares and analyzes the prediction performance of the model. The experimental results show that this method can predict the stock market in a very simple way. At the same time, we use this method to predict the Shanghai stock exchange composite index, and further verify the effectiveness and universality of the method.

## Introduction

Forecasting is an important means of reducing risk and increase revenue in financial sector. Stock price prediction models can be divided into two categories: statistical model and artificial intelligence model. The former models include ANFIS [1], ARIMA [2], ARCH [3], GARCH [4], and so on. In such models, the variables must strictly obey the restrictive assumptions of linear or normal distribution. However, because of the uncertainty and complexity of the stock market, it is difficult to make out a strict normal assumption for a linear prediction model. Wang [5] studied the relationship between stock price and the changing of investors' social network. He established a mathematical model based on fuzzy method. However, such models based on external factors are varying from different stock markets. What is more, other external factors, such as economic environment, policy changing and so on also have great relationship with the fluctuation of a stock market. In fact, historical data can somewhat reflect the internal rule for the evolution of a stock market. Artificial intelligence models can reveal the internal rule and therefore achieve the desired results without any strict assumptions. Such models have better nonlinear processing capabilities, so many researchers have applied it to the prediction of various fields [6–8], such as Mishra [9]use it forecast the PM 2.5 during haze episodes. Raza [10]proposed artificial intelligence method to forecast the load demand of smart grid.

Artificial Neural Networks(ANNs) is a machine learning algorithm that simulates human brain learning, which is suitable for the calculation and prediction of complex systems [11]. ANNs can find the mapping relationship between variables in any precision, and it also has good self-adaptability, self-organization, self-learning ability and generalization ability [12]. These characteristics can meet the demand of the general market trend forecast. Dumitru [13]propose that the prediction method based on ANNs is more suitable for multi-variable prediction, especially for wind market forecasting. John [14]analyzed the characteristics of self-adaptive and self-learning to explore the rules of historical rainfall data. As can be seen from these examples, ANNs are especially suitable for general system prediction in various fields. In stock market forecasting, the back propagation Neural Network(BPNN) models, which is a type of the ANNs, is applied to predict the daily Shanghai Stock Exchange Composite Index [15]. However, when the stock market has complicated situation, for example, the fluctuation is more frequent and the fluctuation amplitude is relatively large, the method expose certain limitations. First of all, the stock market trading is not only non-linear, but also chaotic. Therefore, it is difficult to accurately predict the stock market trend by relying on a single neural network. In addition, due to the uncertainty of stock market fluctuation, ANNs is more prone to overtraining and over-fitting.

The hybrid models which combined ANNs with other approaches have been applied to stock forecasting area due to superior performance than individual models. At present, the comprehensive model of stock market forecasting based on neural network is divided into two aspects; On the one hand, in view of the original noise problem existing in stock market forecast, some researchers attempt to combine the fuzzy set theory with BPNN to reduce the noise of the stock data. Tu [16] proposed the RSEIT2FNN model based on type-2 fuzzy theory and neural network learning algorithm, which combined more recent research achievements on fuzzy theory with neural network. Other scholars established models with fuzzy classification and prediction methods and studied them in more application areas [17–18]. Given the chaotic state of stock market values, Song and Chissom propose the fuzzy time series (FTS) forecasting model [19–21] based on the fuzzy set theory for the first time.Then, other scholars try to combine neural network and fuzzy time series, such as Aladag et al [22]use BPNN to determine fuzzy relations in their fuzzy time series method. The approach combined FTS with ANNs is effective, and that have been widely applied to stock index forecasting [23–24]. But these methods are prone to over-fuzzy, which leads to the reduction of the regular information contained in the original stock market value. On the other hand, for the overtraining and over-fitting of neural network algorithm in the study of stock data, some scholars have tried to combine the stock volatility with BPNN. Wang [25]propose a new approach to forecasting the stock prices via the Wavelet De-noising-based Back Propagation (WDBP) neural network. The model discusses the accuracy of prediction from the Angle of stock market fluctuation, but it does not solve the problem of noise and chaos. Generally speaking, the stock market is influenced by a variety of factors, dynamic, multivariate complex systems, so it is necessary to explore the optimal solution through technology integration. So far, in terms of stock market forecasting, the combination of fuzzy, fluctuating and BPNN synthesis is very rare.

The aim of this paper is to propose a new neural model to improve learning efficiency and predictive power. Therefore, we propose a hybrid forecasting method called High-order-fuzzy-fluctuation-Trends-based Back Propagation(HTBP)neural network modal. In such a model, the original data are first decomposed into multiple layers by the High-Order-Fuzzy-Fluctuation series. The algorithm node is consistent with the order of the wave sequence. This paper is the first attempt to utilize the HTBP based algorithm for forecasting the stock prices. The advantages of the model can be summarized as follows: (1)It combines fuzzy set theory and neural network algorithm to avoid overfitting problems existed in traditional models. (2)BP neural network algorithm can intelligently explore the internal rules of the actual existence of sequential data, without the need to analyze the influence factors of specific rules and the path of action. (3)The hybrid modal can reasonably remove noises from the internal rules by proper fuzzy treatment. The HTBP model is used to predict the stock market from 1997 to 2005 using the TAIEX data set and Shanghai Stock Exchange Composite Index (SHSECI) from 2007 to 2015. Furthermore, the superiority of our model is shown by comparing the HTBP with a traditional model based single BP neural network. we also compare the prediction results with several other existing methods, and conclude that the prediction effect of the model is better than the general prediction model.

The remainder of this paper is organized as follows: Section 2 introduces some research on fuzzy time series and the concept and model of BP neural network. Section 3 describes a prediction method based on BP neural network and fuzzy wave trends and logical relationships. In section 4, the model is used to predict the stock market from 1997 to 2005 using different data set. In section 5, summarize the conclusions and potential problems of future research.

## Preliminaries

### Definition of fuzzy-fluctuation time series (FFTS)

Song and Chissom [19–21]combined fuzzy set theory with time series and presented the definitions of fuzzy time series. In this section, we will extend the fuzzy time series to fuzzy-fluctuation time series (FFTS) and propose the related concepts.

#### Definition 1

Let L = {l_1_, l_2_, …, l_g_} be a fuzzy set in the universe of discourse U; it can be defined by its membership function, μ_L_: U → [0,1], where μ_L_(u_i_) denotes the grade of membership of u_i_, U = {u_1_, u_2_, …u_i_, …, u_l_}.

The fluctuation trends of a stock market can be expressed by a linguistic set L = {l_1_, l_2_, l_3_} = {down, equal, up}. The element l_i_ and its subscript *i* is strictly monotonically increasing [26], so the function can be defined as follows, f: l_i_ = f(i). To preserve all of the given information, the discrete L = {l_1_, l_2_, …, l_g_} also can be extended to a continuous label $\bar{\text{L}}=\{{\text{l}}_{\text{a}}|\text{a}\in \text{R}\}$, which satisfies the above characteristics. $\bar{\text{L}}\prime$ is defined as forecasting value.M is defined as a constant to scale the range of $\bar{\text{S}}(\text{i})$ to facilitate machine learning. $\bar{\text{Q}}(\text{i})$ is defined as the s value after scaling.

#### Definition 2

Let F(t)(t = 1, 2, …, T) be a time series of real numbers, where T is the number of the time series G(t) is defined as a fluctuation time series, where G(t) = F(t) − F(t − 1), (t = 2, 3, …, T). Each element of G(t)can be represented by a fuzzy set S(t)(t = 2, 3, …, T) as defined in Definition 1. Then we call time series G(t) to befuzzified into a fuzzy-fluctuation time series (FFTS) *S(t)*.

#### Definition 3

Let *S(t)*(t = n + 1, n + 2, …, T, n ≥ 1) be a FFTS. If S(t) is determined by S(t − 1), S(t − 2), …, S(t − n), then the fuzzy-fluctuation logical relationship is represented by:

S(t−1),S(t−2),…,S(t−n)→S(t) (1)

and it is called the nth-order fuzzy-fluctuation logical relationship (FFLR) of the fuzzy-fluctuation time series, where S(t − n), …, S(t − 2)S(t − 1) is called the left-hand side(LHS) and S(t) is called the right-hand side(RHS) of the FFLR, and S(k)(k = t, t − 1, t − 2, …, t − n) ∈ L. The fuzzy-fluctuation logical relationship can also be represented by:

$$\text{S}(\text{t}-1),\text{S}(\text{t}-2),\ldots ,\text{S}(\text{t}-\text{n})\to \bar{\text{S}}(\text{t})$$ (2)

$\bar{S}(t)$ is introduced to preserve more information, as described in Definition 1.

$$\bar{\text{Q}}(\text{i}+1)=\bar{\text{S}}(\text{i}+1)/\text{M}$$ (3)

$\bar{Q}(i)$ is introduced to help the Machine learning, as described in Definition 1.

$$\text{S}(\text{t}-1),\text{S}(\text{t}-2),\ldots ,\text{S}(\text{t}-\text{n})\to \bar{\text{Q}}(\text{t})$$ (4)

### Basic concept of BP neural network

BP Neural Network belongs to a hierarchical network with powerful nonlinear processing ability. It doesn't need to know the relationship between the form or the variable of the data distribution. It can spontaneously organize training and learning based on the observed training data. In addition, it establishes a nonlinear mapping between the number of variables and the output. The principle of the network is based on the external feedback of the network, and the weight of the network mapping control variables is realized by adjusting the values of the neural network parameters to minimize errors. Based on BP Neural Network algorithm, we can predict future stock market fluctuations by using algorithms to learn historical fuzzy fluctuations. The model of the activation function is tanh(*x*). Compared to the Sigmoid function, the tanh(*x*) has been optimized to overcome the shortcomings of Sigmoid's not zero-centered. The value range of tanh(*x*) is [–1, 1].

$$\text{tanh}(x)=\frac{e^{x}-e^{-x}}{e^{x}+e^{-x}}$$ (5)

**Fig 1:**
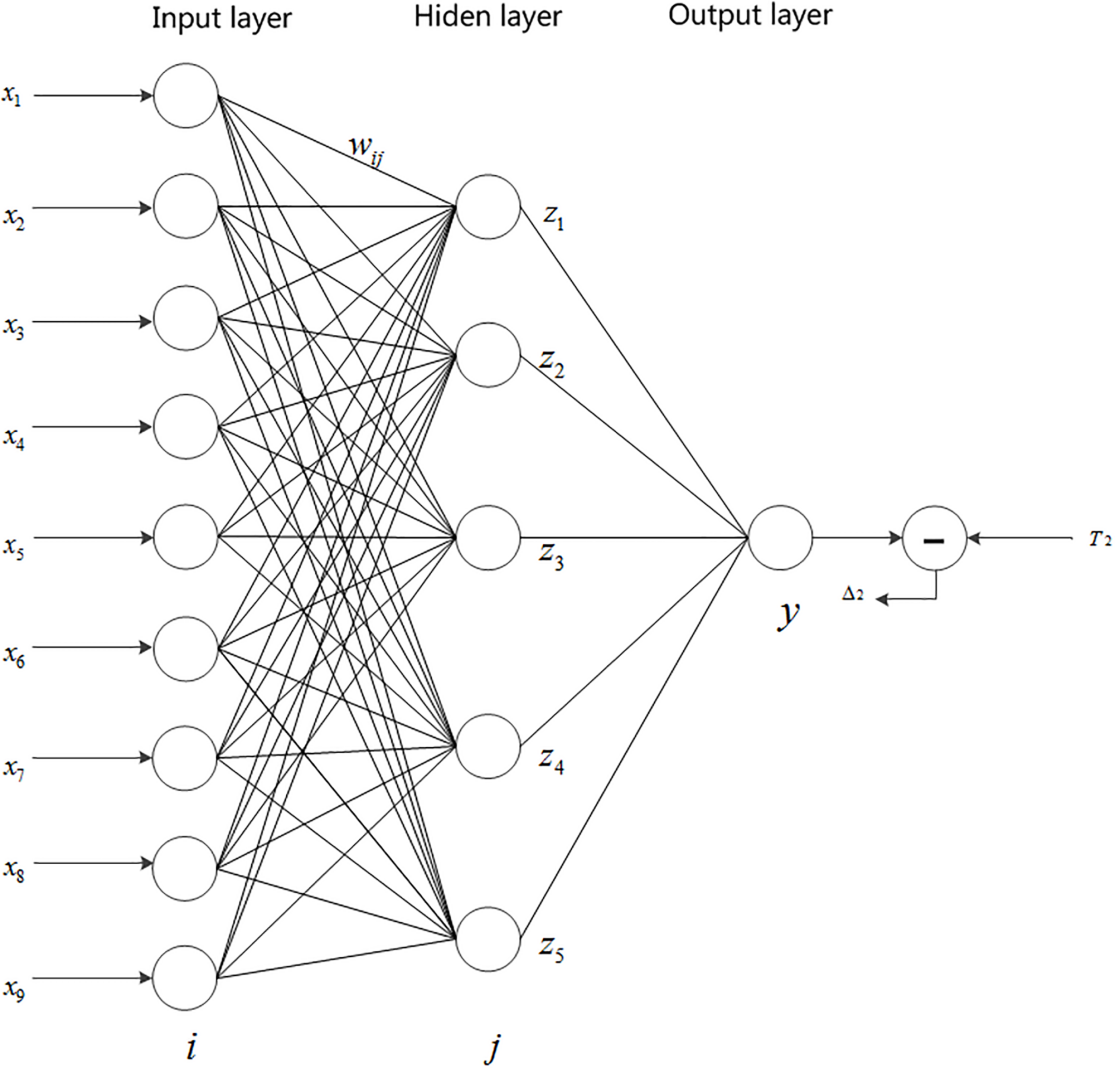
BP neural network structure.

The number of input layer nodes of BP Neural Network model is 9, which denote the 9th-order historical fuzzy-fluctuation trends(Fig 1). The number of output layer nodes of the model is 1, which denote the RHS. When the number of hidden layer nodes is 5, the learning effect is best.

*x*_*i*_ represents the input value for each node of the input layer, and *i* represents the corresponding node number of the input layer. *z*_*j*_ represents the hidden layer node, *w*_*ij*_ represents the weight between input layer and the hidden layer node, and *y*_*j*_ represents the output layer node.

## A novel forecasting model based on BP Neural Network

**Fig 2:**
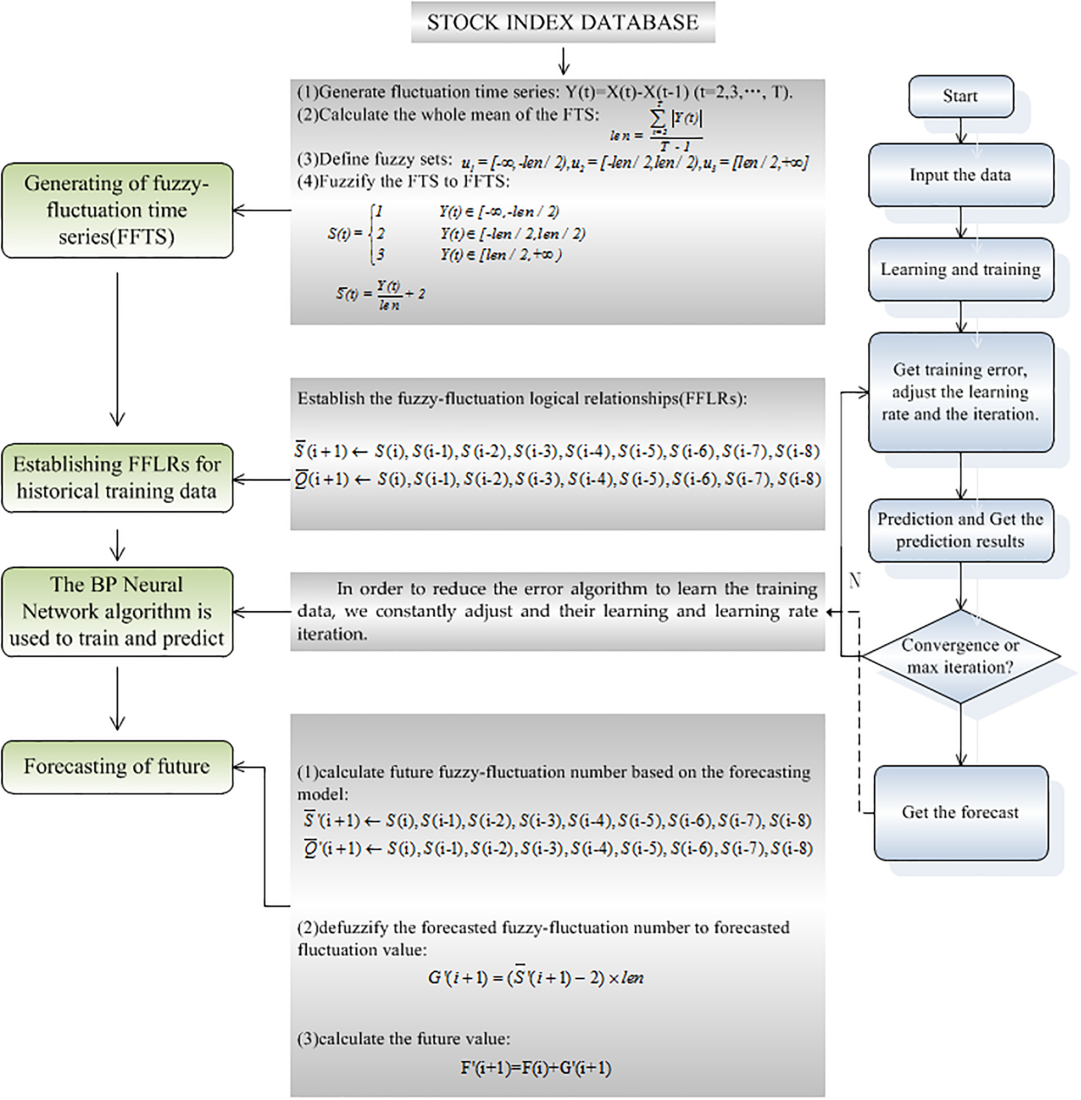
Flowchart of our proposed forecasting model.

In this paper, we propose a novel forecasting model based on High-Order Fuzzy-FluctuationTrends and BP Neural NetworkMachine Learning. In order to compare the forecasting results with other researchers’ work, the authentic TAIEX (Taiwan Stock Exchange Capitalization Weighted Stock Index) is employed to illustrate the forecasting process. The data from January 1999 to October 1999 are used as training time series and the data from November 1999 to December 1999 are used as testing dataset. The basic steps of the proposed model are shown(Fig 2).

**Step 1.** Construct FFTS for historical training data

For each element F(t)(t = 1, 2, …, T) in the historical training time series, its fluctuation trend is determined by G(t) = F(t) − F(t − 1), (t = 2, 3, …, T). According to the range and orientation of the fluctuations, G(t)(t = 2, 3, …, T) can be fuzzified into a linguistic set {down, equal, up}. Let *len* be the whole mean of all elements in the fluctuation time series G(t)(t = 2, 3, …, T), define *u*_1_ = [−∞,−*len*/2), *u*_2_ = [−*len*/2,*len*/2), *u*_3_ = [*len*/2,+∞)], then G(t)(t = 2, 3, …, T) can be fuzzified into a fuzzy-fluctuation time series S(t)(t = 2, 3, …, T).

**Step 2.** Establish nth-order FFLRs for the forecasting model

According to Eq (2), each *S*(*t*)(*t* ≥ *n* + 2) can be represented by its previous n days’ fuzzy-fluctuation number. Therefore, the total of FFLRs for historical training data is *p*_*n*_ = *T* − *n* − 1.

**Step 3.** Determine the parameters for the forecasting model based on BP Neural NetworkMachine Learning algorithm

In this paper, the BP Neural Network method is employed to learnthe fuzzy-fluctuation logical relationship.

$$G\prime (i+1)=(\bar{S}\prime (i+1)-2)\times len$$ (6)

$${\text{F}}^{\prime }(\text{i}+1)=\text{F}(\text{i})+{\text{G}}^{\prime }(\text{i}+1)$$ (7)

**Step 4.** Forecast test time series

For each data in the test time series, its future number can be forecasted according to Eq (7), based on the result of the output of the BP Neural NetworkMachine Learning, its n-order fuzzy-fluctuation trends.

## Empirical analysis

### Forecasting TAIEX

Since lotsofstudiesuseTAIEX1999as an example to illustrate their proposed forecasting methods [27–34]. We also use TAIEX1999 to illustrate the proposed method, and then we compared the accuracy with their models.

**Step 1.** Calculate the fluctuation of each element of the history training dataset. Then, the fluctuation trends will be fuzzified into FFTS by the whole mean of the fluctuation numbers of the training dataset. For example, the whole mean of the historical dataset of TAIEX1999 from January to October is 85. That is to say, *len* = 85. For *F(1)* = 6152.43 and *F(2)* = 6199.91, *G(2)* = 47.48, *S(2)* = 3. In this way, the historical training dataset can be represented by a fuzzified fluctuation dataset as shown in S1 Table.

**Step 2.** Based on the FFTS from 5January 1999 to 30October shown in S1 Table, the nth-order FFLRs for the forecasting model are established as shown in S2 Table. The subscript *I* is used to represent element *l*_*i*_ in the FFLRs for convenience.

For example, suppose n = 6, the 9th-order historical fuzzy-fluctuation trends2,3,1,1,1,2,2,3,3 on 18January 1999 $\bar{S}(11)=0.7524$, then according to Eq (2), the Mapping relationships can be further expressed as:

2,3,1,1,1,2,2,3,3→0.7524

Since parameter $\bar{Q}(11)=\bar{S}(11)/15=0.05016$, then according to Eqs (3) and (4), the Mapping relationships can also be further expressed as:

2,3,1,1,1,2,2,3,3→0.05016

**Step 3.** The detailed BP Neural Network Machine Learning processes are shown in Fig 2.

In order to reduce the error algorithm to learn the training data, we constantly adjust and their learning and learning rate iteration, finally determined the iteration times to 8000 times, learning efficiency is set to 0.00008, the momentum factor is set to 0.003.

**Step 4.** Usethe *FFLR* obtained from historical training data to forecast the test dataset from 1 November 1999 to 30 December.

Firstly, the 9th-order historical fuzzy-fluctuation trends 3,2,2,2,2,3,1,2,2 on 1 November 1999 can be forecasted by the result 0.14506. Therefore, the forecasted fuzzy-fluctuation number is:

$$\bar{S}\prime (i+1)=\bar{Q}\prime (i+1)\times M=0.14506\times 15=2.1759$$

The forecasted fluctuation from current value to next value can be obtained by defuzzifying the fluctuation fuzzy number:

$$G\prime (i+1)=(\bar{S}\prime (i+1)-2)\times len=(2.1759-2)\times 85=14.96$$

Finally, the forecasted value can be obtained by current value and the fluctuation value:

$${\text{F}}^{\prime }(\text{i}+1)=\text{F}(\text{i})+{\text{G}}^{\prime }(\text{i}+1)=7854.85+14.96=7869.81$$

**Fig 3:**
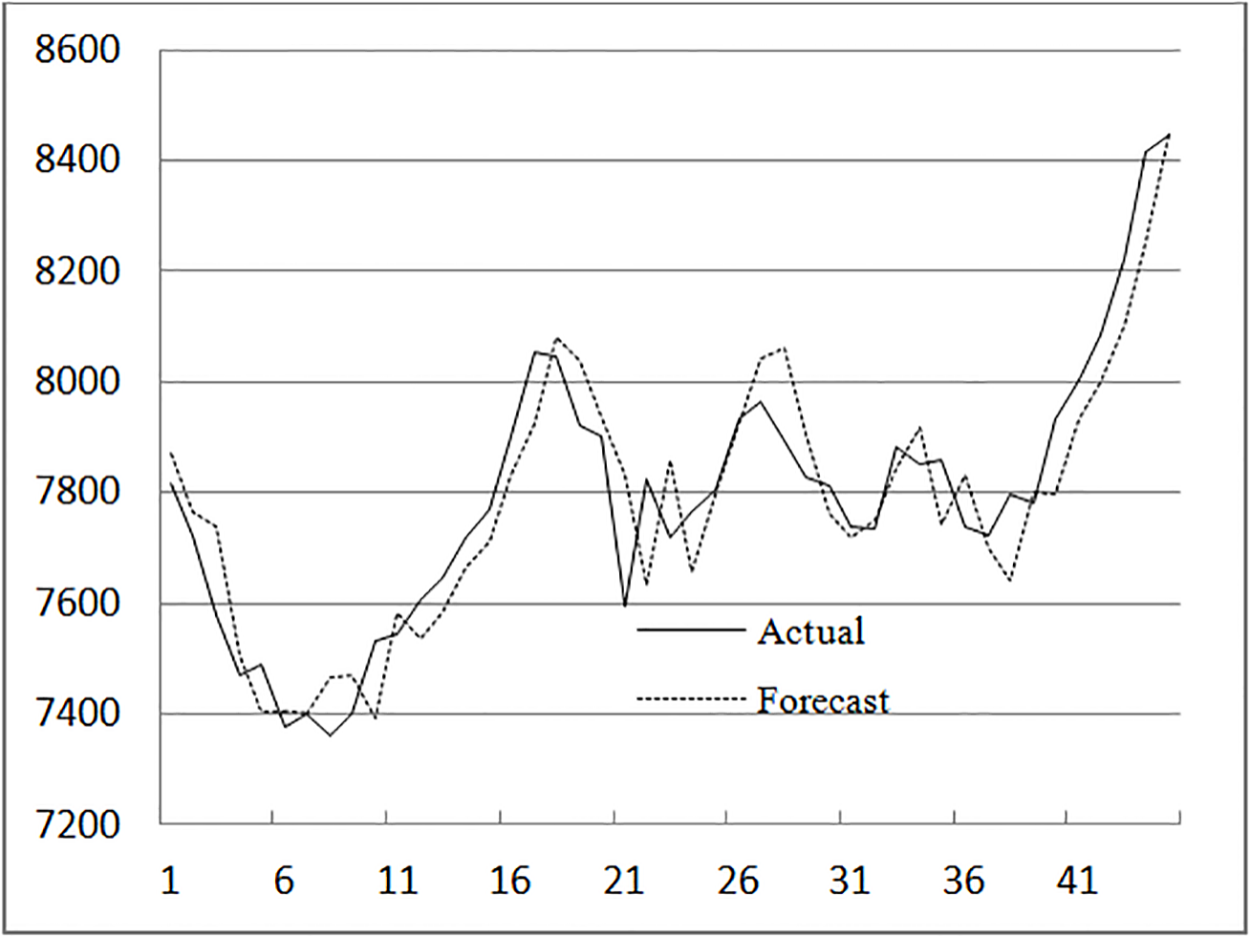
Forecasting results from 1 November1999 to 30 December 1999. Based on the method presented in this paper, the data of 1999 is predicted.

The other forecasting results are shown (Table 1 and Fig 3).

**Table 1:** Forecasting results from 1 November1999 to 30 December 1999.

Date (MM/DD/YYYY)	Actual	Forecast	(Forecast–Actual)^2^	Date (MM/DD/YYYY)	Actual	Forecast	(Forecast–Actual)^2^
11/1/1999	7,814.89	7869.81	3015.71	12/1/1999	7,766.20	7658.73	11550.01
11/2/1999	7,721.59	7767.25	2085.04	12/2/1999	7,806.26	7797.15	83.04
11/3/1999	7,580.09	7737.17	24674.73	12/3/1999	7,933.17	7926.84	40.01
11/4/1999	7,469.23	7505.08	1285.54	12/4/1999	7,964.49	8041.65	5952.94
11/5/1999	7,488.26	7405.53	6844.71	12/6/1999	7,894.46	8061.73	27978.96
11/6/1999	7,376.56	7405.23	821.78	12/7/1999	7,827.05	7907.67	6499.29
11/8/1999	7,401.49	7400.98	0.26	12/8/1999	7,811.02	7761.22	2480.17
11/9/1999	7,362.69	7464.39	10343.58	12/9/1999	7,738.84	7719.02	393.01
11/10/1999	7,401.81	7471.79	4896.83	12/10/1999	7,733.77	7750.31	273.52
11/11/1999	7,532.22	7391.31	19854.75	12/13/1999	7,883.61	7843.78	1586.65
11/15/1999	7,545.03	7581.21	1309.10	12/14/1999	7,850.14	7919.10	4755.54
11/16/1999	7,606.20	7535.24	5034.73	12/15/1999	7,859.89	7744.21	13382.87
11/17/1999	7,645.78	7583.48	3880.77	12/16/1999	7,739.76	7832.19	8542.56
11/18/1999	7,718.06	7665.95	2715.32	12/17/1999	7,723.22	7698.15	628.71
11/19/1999	7,770.81	7711.19	3554.60	12/18/1999	7,797.87	7639.44	25101.33
11/20/1999	7,900.34	7833.44	4475.04	12/20/1999	7,782.94	7801.32	337.75
11/22/1999	8,052.31	7924.00	16463.38	12/21/1999	7,934.26	7796.21	19056.81
11/23/1999	8,046.19	8083.08	1360.55	12/22/1999	8,002.76	7932.06	4999.04
11/24/1999	7,921.85	8037.94	13476.54	12/23/1999	8,083.49	7998.60	7205.82
11/25/1999	7,904.53	7935.50	992.02	12/24/1999	8,219.45	8099.29	14439.44
11/26/1999	7,595.44	7833.93	56879.02	12/27/1999	8,415.07	8252.86	26313.12
11/29/1999	7,823.90	7632.06	36802.23	12/28/1999	8,448.84	8452.34	12.24
11/30/1999	7,720.87	7858.64	18981.79	Root Mean Square Error(RMSE)			96.77

This paper compares the difference between the predicted value and the actual value, and the objective is to evaluate the prediction performance. In the comparison of time series model, the broad indexes are the mean squared error (MSE), root of the mean squared error (RMSE), mean absolute error (MAE), mean percentage error (MPE), etc. These indicators are defined by Eqs (8)–(11):

$$MSE=\frac{\sum_{t=1}^{n}{\left(forecast(t)-actual(t)\right)}^{2}}{n}$$ (8)

$$RMSE=\sqrt{\frac{\sum_{t=1}^{n}{\left(forecast(t)-actual(t)\right)}^{2}}{n}}$$ (9)

$$MAE=\frac{\sum_{t=1}^{n}\left|\left(forecast(t)-actual(t)\right)\right|}{n}$$ (10)

$$MPE=\frac{\sum_{t=1}^{n}\left|\left(forecast(t)-actual(t)\right)\right|/actual(t)}{n}$$ (11)

where *n* denotes the number of values forecasted, *forecast(t)* and *actual(t)* denote the predicted value and actual value at time *t*, respectively. With respect to the proposed method for the 9th-order, the MSE, RMSE, MAE, and MPE are 9363.57, 96.76, 79.54, and 0.01, respectively.

Let the order number *n* vary from 2 to 10, the RMSEs for different nth-order forecasting models are listed in Table 2. The item “Average” refers to the RMSE for the average forecasting results of these different nth-order(n = 2,3,…,10) models.

**Table 2:** Comparison of forecasting errors for different nth-orders.

*n*	2	3	4	5	6	7	8	9	10	Average
RMSE	99.19	98.25	95.50	98.17	94.71	98.95	99.57	96.77	96.88	97.55

**Fig 4:**
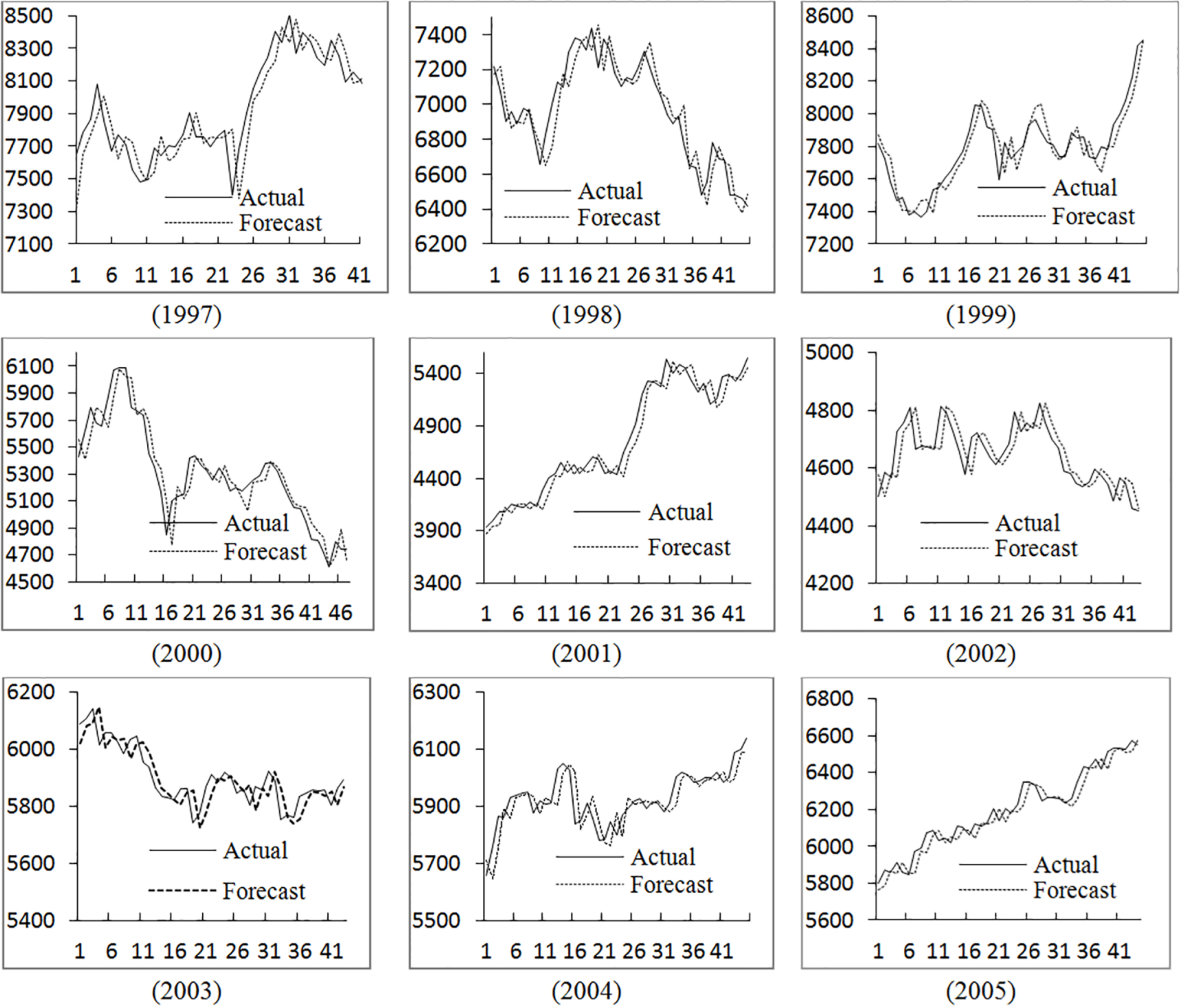
The stock market fluctuation for TAIEX test dataset (1997–2005). Based on the method presented in this paper, the results of Taiwan stock market data from 1999 to 2005 are predicted.

In practical forecasting, the average of results for different nth-order (n = 2,3,…,9) forecasting models is adopted to avoid the uncertainty. The proposed method is employed to forecast the TAIEX from 1997 to 2005. The forecasting results and errors are shown (Fig 4 and Table 3).

**Table 3:** RMSEs of forecast errors for TAIEX 1997 to 2005.

Year	1997	1998	1999	2000	2001	2002	2003	2004	2005
RMSE	142.99	112.51	96.77	126.85	120.12	66.39	54.87	58.10	54.7

Table 4 shows the comparison results for RMSEs of different methods for predicting TAIEX1999. As can be seen from this table, the performance of the proposed method is acceptable. The best advantage of this method is that you do not need to determine the target function, nor do you need to determine the mapping rules. Learn from the algorithm and find the rules. Although some other methods of RMSEs are superior to the methods presented in this article, they usually need to determine complex rules to predict the results. In practice, however, it is often difficult to establish proper rules. The method presented in this paper is very simple and easy to implement computer program.

**Table 4:** A comparison of RMSEs for different methods for forecasting the TAIEX1999.

	Methods	RMSE								
		I	II	III	IV	V	VI	VII	VIII	IX
		1997	1998	1999	2000	2001	2002	2003	2004	2005
A	Chen and Chang’s Method[27]	N	N	**123.64**	131.1	115.08	73.06	66.36	60.48	N
B	Chen and Chen’s Method[28]	N	N	**119.32**	129.87	123.12	71.01	65.14	61.94	N
C	Chen et al.’s Method[39]	N	N	**102.34**	131.25	113.62	65.77	52.23	56.16	N
D	Cheng et al.’s method[30]	N	N	**100.74**	125.62	113.04	62.94	51.46	54.24	N
E	Chen and Kao’s method[31]	N	N	**87.63**	125.34	114.57	76.86	54.29	58.17	N
F	Guan S’s Method[32]	N	N	**101.11**	127.47	114.19	61.92	53.05	53.07	N
G	Jia’s method[33]	143.60	115.34	**99.12**	125.70	115.91	70.43	54.26	57.24	54.68
H	Guan H J’s method[34]	141.89	119.85	**99.03**	128.62	125.64	66.29	53.2	56.11	55.83
I	The proposed	142.99	112.51	**96.77**	126.85	120.12	66.39	54.87	58.10	54.7

### Friedman test

In order to verify the validity of the model proposed in this paper, we applied the Friedman test for the significance test based on JanezDemˇsar’s [35] study. The Friedman test was a non-parametric statistical test proposed by Milton Friedman [36–39]. It sequenced the algorithm of each data set, the best algorithm got the rank 1, and the second best was 2…, as shown in Table 6. Let $r_{i}^{j}$ be the rank of the *j*-th of *k* algorithms on the *i*-th of N data sets. The Friedman testwill compares the average ranks of algorithms, $R_{\text{j}}=\frac{1}{N}\sum_{i}r_{i}^{j}$. the Friedman statisticis distributed according to $\chi_{F}^{2}$ with *k* − 1 degrees of freedom, when *N* and *k* are big enough.

$$\chi_{F}^{2}=\frac{12N}{\text{k}(k+1)}\left[\sum_{j=1}^{k}R_{j}^{2}-\frac{k{\left(k+1\right)}^{2}}{4}\right]$$ (12)

Iman and Davenport [40]thinked that Friedman’s $\chi_{F}^{2}$ is undesirably conservative and proposed a better statistic. Which is distributed according to the F-distribution with *k* − 1 and (*k* − 1)(*N* − 1) degrees of freedom.

$$F_{F}=\frac{(N-1)\chi_{F}^{2}}{N(k-1)-\chi_{F}^{2}}$$ (13)

Nemenyitest [41] is used when compared between all classifiers. The performance of the two classifiers is very different if the corresponding average level is at least different.

$$CD=q_{\alpha }\sqrt{\frac{k(k+1)}{6N}}$$ (14)

This article will rank the data sets from 1999 to 2004 and sort the different methods based on the RMSE error, as shown in Table 5.

**Table 5:** The sorting of different prediction methods based on RMSE for forecasting the TAIEX1999.

	A	B	C	D	E	F	G	H	I
III	123.64(9)	119.32(8)	102.34(7)	100.74(5)	87.63(1)	101.11(6)	99.12(4)	99.03(2)	96.77(3)
IV	131.1(8)	129.87(7)	131.25(9)	125.62(2)	125.34(1)	127.47(5)	125.7(3)	128.62(6)	126.85(4)
V	115.08(5)	123.12(8)	113.62(2)	113.04(1)	114.57(4)	114.19(3)	115.91(6)	125.64(9)	120.12(7)
VI	73.06(8)	71.01(7)	65.77(3)	62.94(2)	76.86(9)	61.92(1)	70.43(6)	66.29(4)	66.39(5)
VII	66.36(9)	65.14(8)	52.23(2)	51.46(1)	54.29(6)	53.05(3)	54.26(5)	53.2(4)	54.87(7)
VIII	60.48(8)	61.94(9)	56.16(4)	54.24(2)	58.17(7)	53.07(1)	57.24(5)	56.11(3)	58.1(6)
average rank	7.83	7.83	4.5	2.17	4.67	3.17	4.83	4.67	5.33

Using the data in Table 5, we can calculate:

$$\begin{array}{cc} \chi_{F}^{2} & =\frac{12\times 6}{9\times 10}\left[7.83^{2}+7.83^{2}+4.5^{2}+2.17^{2}+4.67^{2}+3.17^{2}+4.83^{2}+4.67^{2}+5.33^{2}-\frac{9\times 10^{2}}{4}\right]\\ & =22.38 \end{array}$$

$$\begin{array}{cc} F_{F} & =\frac{5\times 22.38}{6\times 8-22.38}\\ & =4.37 \end{array}$$

With 9 methods and 6 data sets, *F*_*F*_ is distributed according to the F distribution with 9 − 1 = 8 and (9 − 1) × (6 − 1) = 40 degrees of freedom. The critical value of *F*(8,40) for *α* = 0.05 is 2.18, so we reject the null-hypothesis. Next, we used the Nemenyi test for pairwise comparisons. The critical value of CD for *α* = 0.05 is 3.102.

$$\begin{array}{l} CD=3.102\times \sqrt{\frac{9\times (9+1)}{6\times 6}}\\ =4.90 \end{array}$$

According to the average order value in the table, the difference between method A and method B exceeds the critical value, and the others are not exceeded. Therefore, there are significant differences between methods A,B and D(7.83–2.17>4.9), and no significant differences among other algorithms. In general, there is no significant difference between the proposed method and the latest methods in predicting the effect of error and predictive value.

### Forecasting Shanghai Stock Exchange Composite Index

TheSHSECI is China's most typical stock market index. In further research, we apply the method to SHSECI's stock market forecast from 2007 to 2015. We use the real data set of SHSECI's closing price from January to October as training data, and data sets from November to December are used as test data. The RMSEs for the prediction error is shown in Table 6.

**Table 6:** RMSEs of forecast errors for SHSECI from 2007 to 2015.

	Year								
	2007	2008	2009	2010	2011	2012	2013	2014	2015
RMSE	123.89	57.44	48.92	47.34	28.37	25.84	21.43	50.59	59.69

From Table 6, We can see that this method can successfully predict the SHSECI stock market.

## Conclusions

This paper presents a prediction model based on high order fuzzy fluctuation and BP neural network. This method is based on the high order fuzzy logic relation of time series and then uses the self-learning of BPNN to automatically find the optimal prediction rules to predict the fluctuation trend. The greatest advantage of this approach is that the fuzzy theory, stock market fluctuation model and neural network algorithm are combined to construct a new model, which solves the problem of overfitting and over-fuzzy existing models. Experiments show that the parameters generated from the training data set can also be used for future data sets. To compare the performance of other methods, we take TAIEX1999 as an example. We also predicted the validity and universality of TAIEX 1997-2005and Shanghai Stock Exchange Composite Index (SHSECI) from 2007 to 2015. The model presented in this paper has a significant advantage in universality, flexibility and comprehensibility. However, because of the influence of changing external factors, the accuracy of the forecasting results is just acceptable comparing with other models. In further research, we will take more consideration of the influence of external factors to improve the accuracy. Moreover, we will consider other factors that may affect the volatility of the stock market, such as trading volume, starting value, final value, etc. We will also consider the impact of other stock markets, such as the Dow Jones, the NASDAQ, and so on.

## Supporting information

S1 Table Historical training data and fuzzified fluctuation data of TAIEX 1999. (DOCX)

S2 Table The FFLRs for historical training data of TAIEX 1999. (DOCX)
