# Supplementary material for: A novel stock forecasting model based on High-order-fuzzy-fluctuation Trends and Back Propagation Neural Network
Source: PLoS One. 2018 Feb 8;13(2):e0192366. doi: 10.1371/journal.pone.0192366 (PMC5805297; doi:10.1371/journal.pone.0192366)
Supplement: S1 Table — (DOCX) [file pone.0192366.s001.docx]

**S1 Table**

| **Date (MM/DD/YYYY)** | **TAIEX** | **Fluctuation** | **Fuzzified** | **Date (MM/DD/YYYY)** | **TAIEX** | **Fluctuation** | **Fuzzified** | **Date (MM/DD/YYYY)** | **TAIEX** | **Fluctuation** | **Fuzzified** |
| --- | --- | --- | --- | --- | --- | --- | --- | --- | --- | --- | --- |
| 1/5/1999 | 6152.43 | - | - | 4/17/1999 | 7581.5 | 114.68 | 3 | 7/26/1999 | 7595.71 | −128.81 | 1 |
| 1/6/1999 | 6199.91 | 47.48 | 3 | 4/19/1999 | 7623.18 | 41.68 | 2 | 7/27/1999 | 7367.97 | −227.74 | 1 |
| 1/7/1999 | 6404.31 | 204.4 | 3 | 4/20/1999 | 7627.74 | 4.56 | 2 | 7/28/1999 | 7484.5 | 116.53 | 3 |
| 1/8/1999 | 6421.75 | 17.44 | 2 | 4/21/1999 | 7474.16 | −153.58 | 1 | 7/29/1999 | 7359.37 | −125.13 | 1 |
| 1/11/1999 | 6406.99 | −14.76 | 2 | 4/22/1999 | 7494.6 | 20.44 | 2 | 7/30/1999 | 7413.11 | 53.74 | 3 |
| 1/12/1999 | 6363.89 | −43.1 | 1 | 4/23/1999 | 7612.8 | 118.2 | 3 | 7/31/1999 | 7326.75 | −86.36 | 1 |
| 1/13/1999 | 6319.34 | −44.55 | 1 | 4/26/1999 | 7629.09 | 16.29 | 2 | 8/2/1999 | 7195.94 | −130.81 | 1 |
| 1/14/1999 | 6241.32 | −78.02 | 1 | 4/27/1999 | 7550.13 | −78.96 | 1 | 8/3/1999 | 7175.19 | −20.75 | 2 |
| 1/15/1999 | 6454.6 | 213.28 | 3 | 4/28/1999 | 7496.61 | −53.52 | 1 | 8/4/1999 | 7110.8 | −64.39 | 1 |
| 1/16/1999 | 6483.3 | 28.7 | 2 | 4/29/1999 | 7289.62 | −206.99 | 1 | 8/5/1999 | 6959.73 | −151.07 | 1 |
| 1/18/1999 | 6377.25 | −106.05 | 1 | 4/30/1999 | 7371.17 | 81.55 | 3 | 8/6/1999 | 6823.52 | −136.21 | 1 |
| 1/19/1999 | 6343.36 | −33.89 | 2 | 5/3/1999 | 7383.26 | 12.09 | 2 | 8/7/1999 | 7049.74 | 226.22 | 3 |
| 1/20/1999 | 6310.71 | −32.65 | 2 | 5/4/1999 | 7588.04 | 204.78 | 3 | 8/9/1999 | 7028.01 | −21.73 | 2 |
| 1/21/1999 | 6332.2 | 21.49 | 2 | 5/5/1999 | 7572.16 | −15.88 | 2 | 8/10/1999 | 7269.6 | 241.59 | 3 |
| 1/22/1999 | 6228.95 | −103.25 | 1 | 5/6/1999 | 7560.05 | −12.11 | 2 | 8/11/1999 | 7228.68 | −40.92 | 2 |
| 1/25/1999 | 6033.21 | −195.74 | 1 | 5/7/1999 | 7469.33 | −90.72 | 1 | 8/12/1999 | 7330.24 | 101.56 | 3 |
| 1/26/1999 | 6115.64 | 82.43 | 3 | 5/10/1999 | 7484.37 | 15.04 | 2 | 8/13/1999 | 7626.05 | 295.81 | 3 |
| 1/27/1999 | 6138.87 | 23.23 | 2 | 5/11/1999 | 7474.45 | −9.92 | 2 | 8/16/1999 | 8018.47 | 392.42 | 3 |
| 1/28/1999 | 6063.41 | −75.46 | 1 | 5/12/1999 | 7448.41 | −26.04 | 2 | 8/17/1999 | 8083.43 | 64.96 | 3 |
| 1/29/1999 | 5984 | −79.41 | 1 | 5/13/1999 | 7416.2 | −32.21 | 2 | 8/18/1999 | 7993.71 | −89.72 | 1 |
| 1/30/1999 | 5998.32 | 14.32 | 2 | 5/14/1999 | 7592.53 | 176.33 | 3 | 8/19/1999 | 7964.67 | −29.04 | 2 |
| 2/1/1999 | 5862.79 | −135.53 | 1 | 5/15/1999 | 7576.64 | −15.89 | 2 | 8/20/1999 | 8117.42 | 152.75 | 3 |
| 2/2/1999 | 5749.64 | −113.15 | 1 | 5/17/1999 | 7599.76 | 23.12 | 2 | 8/21/1999 | 8153.57 | 36.15 | 2 |
| 2/3/1999 | 5743.86 | −5.78 | 2 | 5/18/1999 | 7585.51 | −14.25 | 2 | 8/23/1999 | 8119.98 | −33.59 | 2 |
| 2/4/1999 | 5514.89 | −228.97 | 1 | 5/19/1999 | 7614.6 | 29.09 | 2 | 8/24/1999 | 7984.39 | −135.59 | 1 |
| 2/5/1999 | 5474.79 | −40.1 | 2 | 5/20/1999 | 7608.88 | −5.72 | 2 | 8/25/1999 | 8127.09 | 142.7 | 3 |
| 2/6/1999 | 5710.18 | 235.39 | 3 | 5/21/1999 | 7606.69 | −2.19 | 2 | 8/26/1999 | 8097.57 | −29.52 | 2 |
| 2/8/1999 | 5822.98 | 112.8 | 3 | 5/24/1999 | 7588.23 | −18.46 | 2 | 8/27/1999 | 8053.97 | −43.6 | 1 |
| 2/9/1999 | 5723.73 | −99.25 | 1 | 5/25/1999 | 7417.03 | −171.2 | 1 | 8/30/1999 | 8071.36 | 17.39 | 2 |
| 2/10/1999 | 5798 | 74.27 | 3 | 5/26/1999 | 7426.63 | 9.6 | 2 | 8/31/1999 | 8157.73 | 86.37 | 3 |
| 2/20/1999 | 6072.33 | 274.33 | 3 | 5/27/1999 | 7469.01 | 42.38 | 2 | 9/1/1999 | 8273.33 | 115.6 | 3 |
| 2/22/1999 | 6313.63 | 241.3 | 3 | 5/28/1999 | 7387.37 | −81.64 | 1 | 9/2/1999 | 8226.15 | −47.18 | 1 |
| 2/23/1999 | 6180.94 | −132.69 | 1 | 5/29/1999 | 7419.7 | 32.33 | 2 | 9/3/1999 | 8073.97 | −152.18 | 1 |
| 2/24/1999 | 6238.87 | 57.93 | 3 | 5/31/1999 | 7316.57 | −103.13 | 1 | 9/4/1999 | 8065.11 | −8.86 | 2 |
| 2/25/1999 | 6275.53 | 36.66 | 2 | 6/1/1999 | 7397.62 | 81.05 | 3 | 9/6/1999 | 8130.28 | 65.17 | 3 |
| 2/26/1999 | 6318.52 | 42.99 | 3 | 6/2/1999 | 7488.03 | 90.41 | 3 | 9/7/1999 | 7945.76 | −184.52 | 1 |
| 3/1/1999 | 6312.25 | −6.27 | 2 | 6/3/1999 | 7572.91 | 84.88 | 3 | 9/8/1999 | 7973.3 | 27.54 | 2 |
| 3/2/1999 | 6263.54 | −48.71 | 1 | 6/4/1999 | 7590.44 | 17.53 | 2 | 9/9/1999 | 8025.02 | 51.72 | 3 |
| 3/3/1999 | 6403.14 | 139.6 | 3 | 6/5/1999 | 7639.3 | 48.86 | 3 | 9/10/1999 | 8161.46 | 136.44 | 3 |
| 3/4/1999 | 6393.74 | −9.4 | 2 | 6/7/1999 | 7802.69 | 163.39 | 3 | 9/13/1999 | 8178.69 | 17.23 | 2 |
| 3/5/1999 | 6383.09 | −10.65 | 2 | 6/8/1999 | 7892.13 | 89.44 | 3 | 9/14/1999 | 8092.02 | −86.67 | 1 |
| 3/6/1999 | 6421.73 | 38.64 | 2 | 6/9/1999 | 7957.71 | 65.58 | 3 | 9/15/1999 | 7971.04 | −120.98 | 1 |
| 3/8/1999 | 6431.96 | 10.23 | 2 | 6/10/1999 | 7996.76 | 39.05 | 2 | 9/16/1999 | 7968.9 | −2.14 | 2 |
| 3/9/1999 | 6493.43 | 61.47 | 3 | 6/11/1999 | 7979.4 | −17.36 | 2 | 9/17/1999 | 7916.92 | −51.98 | 1 |
| 3/10/1999 | 6486.61 | −6.82 | 2 | 6/14/1999 | 7973.58 | −5.82 | 2 | 9/18/1999 | 8016.93 | 100.01 | 3 |
| 3/11/1999 | 6436.8 | −49.81 | 1 | 6/15/1999 | 7960 | −13.58 | 2 | 9/20/1999 | 7972.14 | −44.79 | 1 |
| 3/12/1999 | 6462.73 | 25.93 | 2 | 6/16/1999 | 8059.02 | 99.02 | 3 | 9/27/1999 | 7759.93 | −212.21 | 1 |
| 3/15/1999 | 6598.32 | 135.59 | 3 | 6/17/1999 | 8274.36 | 215.34 | 3 | 9/28/1999 | 7577.85 | −182.08 | 1 |
| 3/16/1999 | 6672.23 | 73.91 | 3 | 6/21/1999 | 8413.48 | 139.12 | 3 | 9/29/1999 | 7615.45 | 37.6 | 2 |
| 3/17/1999 | 6757.07 | 84.84 | 3 | 6/22/1999 | 8608.91 | 195.43 | 3 | 9/30/1999 | 7598.79 | −16.66 | 2 |
| 3/18/1999 | 6895.01 | 137.94 | 3 | 6/23/1999 | 8492.32 | −116.59 | 1 | 10/1/1999 | 7694.99 | 96.2 | 3 |
| 3/19/1999 | 6997.29 | 102.28 | 3 | 6/24/1999 | 8589.31 | 96.99 | 3 | 10/2/1999 | 7659.55 | −35.44 | 2 |
| 3/20/1999 | 6993.38 | −3.91 | 2 | 6/25/1999 | 8265.96 | −323.35 | 1 | 10/4/1999 | 7685.48 | 25.93 | 2 |
| 3/22/1999 | 7043.23 | 49.85 | 3 | 6/28/1999 | 8281.45 | 15.49 | 2 | 10/5/1999 | 7557.01 | −128.47 | 1 |
| 3/23/1999 | 6945.48 | −97.75 | 1 | 6/29/1999 | 8514.27 | 232.82 | 3 | 10/6/1999 | 7501.63 | −55.38 | 1 |
| 3/24/1999 | 6889.42 | −56.06 | 1 | 6/30/1999 | 8467.37 | −46.9 | 1 | 10/7/1999 | 7612 | 110.37 | 3 |
| 3/25/1999 | 6941.38 | 51.96 | 3 | 7/2/1999 | 8572.09 | 104.72 | 3 | 10/8/1999 | 7552.98 | −59.02 | 1 |
| 3/26/1999 | 7033.25 | 91.87 | 3 | 7/3/1999 | 8563.55 | −8.54 | 2 | 10/11/1999 | 7607.11 | 54.13 | 3 |
| 3/29/1999 | 6901.68 | −131.57 | 1 | 7/5/1999 | 8593.35 | 29.8 | 2 | 10/12/1999 | 7835.37 | 228.26 | 3 |
| 3/30/1999 | 6898.66 | −3.02 | 2 | 7/6/1999 | 8454.49 | −138.86 | 1 | 10/13/1999 | 7836.94 | 1.57 | 2 |
| 3/31/1999 | 6881.72 | −16.94 | 2 | 7/7/1999 | 8470.07 | 15.58 | 2 | 10/14/1999 | 7879.91 | 42.97 | 3 |
| 4/1/1999 | 7018.68 | 136.96 | 3 | 7/8/1999 | 8592.43 | 122.36 | 3 | 10/15/1999 | 7819.09 | −60.82 | 1 |
| 4/2/1999 | 7232.51 | 213.83 | 3 | 7/9/1999 | 8550.27 | −42.16 | 2 | 10/16/1999 | 7829.39 | 10.3 | 2 |
| 4/3/1999 | 7182.2 | −50.31 | 1 | 7/12/1999 | 8463.9 | −86.37 | 1 | 10/18/1999 | 7745.26 | −84.13 | 1 |
| 4/6/1999 | 7163.99 | −18.21 | 2 | 7/13/1999 | 8204.5 | −259.4 | 1 | 10/19/1999 | 7692.96 | −52.3 | 1 |
| 4/7/1999 | 7135.89 | −28.1 | 2 | 7/14/1999 | 7888.66 | −315.84 | 1 | 10/20/1999 | 7666.64 | −26.32 | 2 |
| 4/8/1999 | 7273.41 | 137.52 | 3 | 7/15/1999 | 7918.04 | 29.38 | 2 | 10/21/1999 | 7654.9 | −11.74 | 2 |
| 4/9/1999 | 7265.7 | −7.71 | 2 | 7/16/1999 | 7411.58 | −506.46 | 1 | 10/22/1999 | 7559.63 | −95.27 | 1 |
| 4/12/1999 | 7242.4 | −23.3 | 2 | 7/17/1999 | 7366.23 | −45.35 | 1 | 10/25/1999 | 7680.87 | 121.24 | 3 |
| 4/13/1999 | 7337.85 | 95.45 | 3 | 7/19/1999 | 7386.89 | 20.66 | 2 | 10/26/1999 | 7700.29 | 19.42 | 2 |
| 4/14/1999 | 7398.65 | 60.8 | 3 | 7/20/1999 | 7806.85 | 419.96 | 3 | 10/27/1999 | 7701.22 | 0.93 | 2 |
| 4/15/1999 | 7498.17 | 99.52 | 3 | 7/21/1999 | 7786.65 | −20.2 | 2 | 10/28/1999 | 7681.85 | −19.37 | 2 |
| 4/16/1999 | 7466.82 | −31.35 | 2 | 7/22/1999 | 7678.67 | −107.98 | 1 | 10/29/1999 | 7706.67 | 24.82 | 2 |
| 4/17/1999 | 7581.5 | 114.68 | 3 | 7/23/1999 | 7724.52 | 45.85 | 3 | 10/30/1999 | 7854.85 | 148.18 | 3 |
