# Supplementary material for: A novel stock forecasting model based on High-order-fuzzy-fluctuation Trends and Back Propagation Neural Network
Source: PLoS One. 2018 Feb 8;13(2):e0192366. doi: 10.1371/journal.pone.0192366 (PMC5805297; doi:10.1371/journal.pone.0192366)
Supplement: S2 Table — (DOCX) [file pone.0192366.s002.docx]

**S2 Table**

| **Date (MM/DD/YYYY)** | **FFLR** | **Date (MM/DD/YYYY)** | **FFLR** | **Date (MM/DD/YYYY)** | **FFLR** | **Date (MM/DD/YYYY)** | **FFLR** | |
| --- | --- | --- | --- | --- | --- | --- | --- | --- |
| 1999/1/18 | 2,3,1,1,1,2,2,3,3→0.05016  1,2,3,1,1,1,2,2,3→0.10675  2,1,2,3,1,1,1,2,2→0.10773  2,2,1,2,3,1,1,1,2→0.15019  2,2,2,1,2,3,1,1,1→0.05235  1,2,2,2,1,2,3,1,1→-0.02019  1,1,2,2,2,1,2,3,1→0.19798  3,1,1,2,2,2,1,2,3→0.15155  2,3,1,1,2,2,2,1,2→0.07415  1,2,3,1,1,2,2,2,1→0.07105  1,1,2,3,1,1,2,2,2→0.14456  2,1,1,2,3,1,1,2,2→0.02704  1,2,1,1,2,3,1,1,2→0.04459  1,1,2,1,1,2,3,1,1→0.12880  2,1,1,2,1,1,2,3,1→-0.04625  1,2,1,1,2,1,1,2,3→0.10188  2,1,2,1,1,2,1,1,2→0.31795  3,2,1,2,1,1,2,1,1→0.22180  3,3,2,1,2,1,1,2,1→0.05549  1,3,3,2,1,2,1,1,2→0.19158  3,1,3,3,2,1,2,1,1→0.34849  3,3,1,3,3,2,1,2,1→0.32259  3,3,3,1,3,3,2,1,2→0.02926  1,3,3,3,1,3,3,2,1→0.17877  3,1,3,3,3,1,3,3,2→0.16209  2,3,1,3,3,3,1,3,3→0.16705  3,2,3,1,3,3,3,1,3→0.12842  2,3,2,3,1,3,3,3,1→0.09513  1,2,3,2,3,1,3,3,3→0.24282  3,1,2,3,2,3,1,3,3→0.12596  2,3,1,2,3,2,3,1,3→0.12498  2,2,3,1,2,3,2,3,1→0.16364  2,2,2,3,1,2,3,2,3→0.14136  2,2,2,2,3,1,2,3,2→0.18155 | 1999/4/3 | 3,3,2,2,1,3,3,1,1→0.09387  1,3,3,2,2,1,3,3,1→0.11905  2,1,3,3,2,2,1,3,3→0.11129  2,2,1,3,3,2,2,1,3→0.24119  3,2,2,1,3,3,2,2,1→0.12729  2,3,2,2,1,3,3,2,2→0.11506  2,2,3,2,2,1,3,3,2→0.20820  3,2,2,3,2,2,1,3,3→0.18102  3,3,2,2,3,2,2,1,3→0.21139  3,3,3,2,2,3,2,2,1→0.10875  2,3,3,3,2,2,3,2,2→0.22328  3,2,3,3,3,2,2,3,2→0.16602  2,3,2,3,3,3,2,2,3→0.13691  2,2,3,2,3,3,3,2,2→0.01288  1,2,2,3,2,3,3,3,2→0.14936  2,1,2,2,3,2,3,3,3→0.22604  3,2,1,2,2,3,2,3,3→0.14611  2,3,2,1,2,2,3,2,3→0.07140  1,2,3,2,1,2,2,3,2→0.09136  1,1,2,3,2,1,2,2,3→-0.02901  1,1,1,2,3,2,1,2,2→0.19729  3,1,1,1,2,3,2,1,2→0.14282  2,3,1,1,1,2,3,2,1→0.29395  3,2,3,1,1,1,2,3,2→0.12088  2,3,2,3,1,1,1,2,3→0.12384  2,2,3,2,3,1,1,1,2→0.06218  1,2,2,3,2,3,1,1,1→0.14513  2,1,2,2,3,2,3,1,1→0.12555  2,2,1,2,2,3,2,3,1→0.11291  2,2,2,1,2,2,3,2,3→0.10807  2,2,2,2,1,2,2,3,2→0.27163  3,2,2,2,2,1,2,2,3→0.12087  2,3,2,2,2,2,1,2,2→0.15147  2,2,3,2,2,2,2,1,2→0.12216 | 1999/6/11 | 2,3,3,3,3,2,3,3,3→0.11972  2,2,3,3,3,3,2,3,3→0.12877  2,2,2,3,3,3,3,2,3→0.12268  2,2,2,2,3,3,3,3,2→0.21100  3,2,2,2,2,3,3,3,3→0.30223  3,3,2,2,2,2,3,3,3→0.24245  3,3,3,2,2,2,2,3,3→0.28661  3,3,3,3,2,2,2,2,3→0.04189  1,3,3,3,3,2,2,2,2→0.20940  3,1,3,3,3,3,2,2,2→-0.12027  1,3,1,3,3,3,3,2,2→0.14548  2,1,3,1,3,3,3,3,2→0.31594  3,2,1,3,1,3,3,3,3→0.09655  1,3,2,1,3,1,3,3,3→0.21547  3,1,3,2,1,3,1,3,3→0.12664  2,3,1,3,2,1,3,1,3→0.15671  2,2,3,1,3,2,1,3,1→0.02442  1,2,2,3,1,3,2,1,3→0.14555  2,1,2,2,3,1,3,2,1→0.22930  3,2,1,2,2,3,1,3,2→0.10027  2,3,2,1,2,2,3,1,3→0.06559  1,2,3,2,1,2,2,3,1→-0.07012  1,1,2,3,2,1,2,2,3→-0.11438  1,1,1,2,3,2,1,2,2→0.15638  2,1,1,1,2,3,2,1,2→-0.26389  1,2,1,1,1,2,3,2,1→0.09776  1,1,2,1,1,1,2,3,2→0.14954  2,1,1,2,1,1,1,2,3→0.46271  3,2,1,1,2,1,1,1,2→0.11749  2,3,2,1,1,2,1,1,1→0.04864  1,2,3,2,1,1,2,1,1→0.16929  3,1,2,3,2,1,1,2,1→0.03231  1,3,1,2,3,2,1,1,2→-0.04529  1,1,3,1,2,3,2,1,1→0.22473 | 1999/8/21 | 3,2,1,3,3,3,3,2,3→0.16169  2,3,2,1,3,3,3,3,2→0.10699  2,2,3,2,1,3,3,3,3→0.02699  1,2,2,3,2,1,3,3,3→0.24525  3,1,2,2,3,2,1,3,3→0.11018  2,3,1,2,2,3,2,1,3→0.09914  1,2,3,1,2,2,3,2,1→0.14697  2,1,2,3,1,2,2,3,2→0.20107  3,2,1,2,3,1,2,2,3→0.22400  3,3,2,1,2,3,1,2,2→0.09633  1,3,3,2,1,2,3,1,2→0.01398  1,1,3,3,2,1,2,3,1→0.12638  2,1,1,3,3,2,1,2,3→0.18445  3,2,1,1,3,3,2,1,2→-0.01139  1,3,2,1,1,3,3,2,1→0.15493  2,1,3,2,1,1,3,3,2→0.17390  3,2,1,3,2,1,1,3,3→0.24035  3,3,2,1,3,2,1,1,3→0.14685  2,3,3,2,1,3,2,1,1→0.06536  1,2,3,3,2,1,3,2,1→0.03845  1,1,2,3,3,2,1,3,2→0.13165  2,1,1,2,3,3,2,1,3→0.09256  1,2,1,1,2,3,3,2,1→0.21177  3,1,2,1,1,2,3,3,2→0.09820  1,3,1,2,1,1,2,3,3→-0.03311  1,1,3,1,2,1,1,2,3→-0.00947  1,1,1,3,1,2,1,1,2→0.16282  2,1,1,1,3,1,2,1,1→0.12027  2,2,1,1,1,3,1,2,1→0.20878  3,2,2,1,1,1,3,1,2→0.10554  2,3,2,2,1,1,1,3,1→0.15367  2,2,3,2,2,1,1,1,3→0.03257  1,2,2,3,2,2,1,1,1→0.08990  1,1,2,2,3,2,2,1,1→0.21990 | |
| 1999/1/19 |  | 1999/4/6 |  | 1999/6/14 |  | 1999/8/23 |  |  |
| 1999/1/20 |  | 1999/4/7 |  | 1999/6/15 |  | 1999/8/24 |  |  |
| 1999/1/21 |  | 1999/4/8 |  | 1999/6/16 |  | 1999/8/25 |  |  |
| 1999/1/22 |  | 1999/4/9 |  | 1999/6/17 |  | 1999/8/26 |  |  |
| 1999/1/25 |  | 1999/4/12 |  | 1999/6/21 |  | 1999/8/27 |  |  |
| 1999/1/26 |  | 1999/4/13 |  | 1999/6/22 |  | 1999/8/30 |  |  |
| 1999/1/27 |  | 1999/4/14 |  | 1999/6/23 |  | 1999/8/31 |  |  |
| 1999/1/28 |  | 1999/4/15 |  | 1999/6/24 |  | 1999/9/1 |  |  |
| 1999/1/29 |  | 1999/4/16 |  | 1999/6/25 |  | 1999/9/2 |  |  |
| 1999/1/30 |  | 1999/4/17 |  | 1999/6/28 |  | 1999/9/3 |  |  |
| 1999/2/1 |  | 1999/4/19 |  | 1999/6/29 |  | 1999/9/4 |  |  |
| 1999/2/2 |  | 1999/4/20 |  | 1999/6/30 |  | 1999/9/6 |  |  |
| 1999/2/3 |  | 1999/4/21 |  | 1999/7/2 |  | 1999/9/7 |  |  |
| 1999/2/4 |  | 1999/4/22 |  | 1999/7/3 |  | 1999/9/8 |  |  |
| 1999/2/5 |  | 1999/4/23 |  | 1999/7/5 |  | 1999/9/9 |  |  |
| 1999/2/6 |  | 1999/4/26 |  | 1999/7/6 |  | 1999/9/10 |  |  |
| 1999/2/8 |  | 1999/4/27 |  | 1999/7/7 |  | 1999/9/13 |  |  |
| 1999/2/9 |  | 1999/4/28 |  | 1999/7/8 |  | 1999/9/14 |  |  |
| 1999/2/10 |  | 1999/4/29 |  | 1999/7/9 |  | 1999/9/15 |  |  |
| 1999/2/20 |  | 1999/4/30 |  | 1999/7/12 |  | 1999/9/16 |  |  |
| 1999/2/22 |  | 1999/5/3 |  | 1999/7/13 |  | 1999/9/17 |  |  |
| 1999/2/23 |  | 1999/5/4 |  | 1999/7/14 |  | 1999/9/18 |  |  |
| 1999/2/24 |  | 1999/5/5 |  | 1999/7/15 |  | 1999/9/20 |  |  |
| 1999/2/25 |  | 1999/5/6 |  | 1999/7/16 |  | 1999/9/27 |  |  |
| 1999/2/26 |  | 1999/5/7 |  | 1999/7/17 |  | 1999/9/28 |  |  |
| 1999/3/1 |  | 1999/5/10 |  | 1999/7/19 |  | 1999/9/29 |  |  |
| 1999/3/2 |  | 1999/5/11 |  | 1999/7/20 |  | 1999/9/30 |  |  |
| 1999/3/3 |  | 1999/5/12 |  | 1999/7/21 |  | 1999/10/1 |  |  |
| 1999/3/4 |  | 1999/5/13 |  | 1999/7/22 |  | 1999/10/2 |  |  |
| 1999/3/5 |  | 1999/5/14 |  | 1999/7/23 |  | 1999/10/4 |  |  |
| 1999/3/6 |  | 1999/5/15 |  | 1999/7/26 |  | 1999/10/5 |  |  |
| 1999/3/8 |  | 1999/5/17 |  | 1999/7/27 |  | 1999/10/6 |  |  |
| 1999/3/9 |  | 1999/5/18 |  | 1999/7/28 |  | 1999/10/7 |  |  |
| 1999/3/10 | 3,2,2,2,2,3,1,2,3→0.12798  2,3,2,2,2,2,3,1,2→0.09427  1,2,3,2,2,2,2,3,1→0.15367  2,1,2,3,2,2,2,2,3→0.23968  3,2,1,2,3,2,2,2,2→0.19130  3,3,2,1,2,3,2,2,2→0.19987  3,3,3,2,1,2,3,2,2→0.24152  3,3,3,3,2,1,2,3,2→0.21355  3,3,3,3,3,2,1,2,3→0.13027  2,3,3,3,3,3,2,1,2→0.17243  3,2,3,3,3,3,3,2,1→0.05667  1,3,2,3,3,3,3,3,2→0.08936  1,1,3,2,3,3,3,3,3→0.17409  3,1,1,3,2,3,3,3,3→0.20539  3,3,1,1,3,2,3,3,3→0.03014  1,3,3,1,1,3,2,3,3→0.13096  2,1,3,3,1,1,3,2,3→0.12005  2,2,1,3,3,1,1,3,2→0.24075  3,2,2,1,3,3,1,1,3→0.30104 | 1999/5/19 | 2,2,2,3,2,2,2,2,1→0.15615  2,2,2,2,3,2,2,2,2→0.12885  2,2,2,2,2,3,2,2,2→0.13162  2,2,2,2,2,2,3,2,2→0.11885  2,2,2,2,2,2,2,3,2→-0.00094  1,2,2,2,2,2,2,2,3→0.14086  2,1,2,2,2,2,2,2,2→0.16657  2,2,1,2,2,2,2,2,2→0.06930  1,2,2,1,2,2,2,2,2→0.15869  2,1,2,2,1,2,2,2,2→0.05245  1,2,1,2,2,1,2,2,2→0.19690  3,1,2,1,2,2,1,2,2→0.20424  3,3,1,2,1,2,2,1,2→0.19991  3,3,3,1,2,1,2,2,1→0.14708  2,3,3,3,1,2,1,2,2→0.17165  3,2,3,3,3,1,2,1,2→0.26148  3,3,2,3,3,3,1,2,1→0.20348  3,3,3,2,3,3,3,1,2→0.18477  3,3,3,3,2,3,3,3,1→0.16396 | 1999/7/29 | 3,1,1,3,1,2,3,2,1→0.03519  1,3,1,1,3,1,2,3,2→0.17548  3,1,3,1,1,3,1,2,3→0.06560  1,3,1,3,1,1,3,1,2→0.03074  1,1,3,1,3,1,1,3,1→0.11706  2,1,1,3,1,3,1,1,3→0.08283  1,2,1,1,3,1,3,1,1→0.01485  1,1,2,1,1,3,1,3,1→0.02650  1,1,1,2,1,1,3,1,3→0.31076  3,1,1,1,2,1,1,3,1→0.11629  2,3,1,1,1,2,1,1,3→0.32282  3,2,3,1,1,1,2,1,1→0.10124  2,3,2,3,1,1,1,2,1→0.21299  3,2,3,2,3,1,1,1,2→0.36534  3,3,2,3,2,3,1,1,1→0.44111  3,3,3,2,3,2,3,1,1→0.18428  3,3,3,3,2,3,2,3,1→0.06296  1,3,3,3,3,2,3,2,3→0.11056  2,1,3,3,3,3,2,3,2→0.25314 | 1999/10/8 | 3,1,1,2,2,3,2,2,1→0.08704  1,3,1,1,2,2,3,2,2→0.17579  3,1,3,1,1,2,2,3,2→0.31236  3,3,1,3,1,1,2,2,3→0.13456  2,3,3,1,3,1,1,2,2→0.16704  3,2,3,3,1,3,1,1,2→0.08563  1,3,2,3,3,1,3,1,1→0.14141  2,1,3,2,3,3,1,3,1→0.06735  1,2,1,3,2,3,3,1,3→0.09231  1,1,2,1,3,2,3,3,1→0.11269  2,1,1,2,1,3,2,3,3→0.12413  2,2,1,1,2,1,3,2,3→0.05861  1,2,2,1,1,2,1,3,2→0.22842  3,1,2,2,1,1,2,1,3→0.14856  2,3,1,2,2,1,1,2,1→0.13406  2,2,3,1,2,2,1,1,2→0.11814  2,2,2,3,1,2,2,1,1→0.15280  2,2,2,2,3,1,2,2,1→0.24955 | |
| 1999/3/11 |  | 1999/5/20 |  | 1999/7/30 |  | 1999/10/11 |  |  |
| 1999/3/12 |  | 1999/5/21 |  | 1999/7/31 |  | 1999/10/12 |  |  |
| 1999/3/15 |  | 1999/5/24 |  | 1999/8/2 |  | 1999/10/13 |  |  |
| 1999/3/16 |  | 1999/5/25 |  | 1999/8/3 |  | 1999/10/14 |  |  |
| 1999/3/17 |  | 1999/5/26 |  | 1999/8/4 |  | 1999/10/15 |  |  |
| 1999/3/18 |  | 1999/5/27 |  | 1999/8/5 |  | 1999/10/16 |  |  |
| 1999/3/19 |  | 1999/5/28 |  | 1999/8/6 |  | 1999/10/18 |  |  |
| 1999/3/20 |  | 1999/5/29 |  | 1999/8/7 |  | 1999/10/19 |  |  |
| 1999/3/22 |  | 1999/5/31 |  | 1999/8/9 |  | 1999/10/20 |  |  |
| 1999/3/23 |  | 1999/6/1 |  | 1999/8/10 |  | 1999/10/21 |  |  |
| 1999/3/24 |  | 1999/6/2 |  | 1999/8/11 |  | 1999/10/22 |  |  |
| 1999/3/25 |  | 1999/6/3 |  | 1999/8/12 |  | 1999/10/25 |  |  |
| 1999/3/26 |  | 1999/6/4 |  | 1999/8/13 |  | 1999/10/26 |  |  |
| 1999/3/29 |  | 1999/6/5 |  | 1999/8/16 |  | 1999/10/27 |  |  |
| 1999/3/30 |  | 1999/6/7 |  | 1999/8/17 |  | 1999/10/28 |  |  |
| 1999/3/31 |  | 1999/6/8 |  | 1999/8/18 |  | 1999/10/29 |  |  |
| 1999/4/1 |  | 1999/6/9 |  | 1999/8/19 |  | 1999/10/30 |  |  |
| 1999/4/2 |  | 1999/6/10 |  | 1999/8/20 |  | - | - | - |
